# Quality of Life within Horse Welfare Assessment Tools: Informing Decisions for Chronically Ill and Geriatric Horses

**DOI:** 10.3390/ani12141822

**Published:** 2022-07-17

**Authors:** Mariessa Long, Christian Dürnberger, Florien Jenner, Zsófia Kelemen, Ulrike Auer, Herwig Grimm

**Affiliations:** 1 Unit of Ethics and Human-Animal Studies, Messerli Research Institute, University of Veterinary Medicine Vienna, Medical University of Vienna, University of Vienna, Veterinaerplatz 1, 1210 Vienna, Austria; christian.duernberger@vetmeduni.ac.at (C.D.); herwig.grimm@vetmeduni.ac.at (H.G.); 2 Equine Surgery Unit, University Equine Hospital, Department of Companion Animals and Horses, University of Veterinary Medicine Vienna, Veterinaerplatz 1, 1210 Vienna, Austria; florien.jenner@vetmeduni.ac.at (F.J.); zsofia.kelemen@vetmeduni.ac.at (Z.K.); 3 Anaesthesiology and Perioperative Intensive Care Medicine Unit, Department of Companion Animals and Horses, University of Veterinary Medicine Vienna, Veterinaerplatz 1, 1210 Vienna, Austria; ulrike.auer@vetmeduni.ac.at

**Keywords:** quality of life, welfare assessment tools, horses

## Abstract

**Simple Summary:**

Equine Quality of Life is an important concern in decision making in veterinary medicine and is especially relevant for chronically ill or aged horses when euthanasia is considered. To our knowledge, there is no assessment tool for chronically ill or aged horses that assesses equine Quality of Life defined as the horse’s evaluation of their life. However, tools exist to assess equine welfare in different contexts. Therefore, this study aimed to analyse how equine welfare, Quality of Life, well-being and happiness assessment tools define and attempt to measure these concepts. We discuss the tools’ suitability to assess equine Quality of Life in the context of end-of-life decisions for chronically ill or aged horses. Fourteen publications were found via a systematic literature search, describing ten equine welfare assessment tools and one approach to assessing equine Quality of Life in veterinary practice. Some of these welfare assessment tools have the potential to inform the development of a Quality-of-Life assessment tool supporting well-considered decision making towards the end of horses’ lives if they are adjusted to focus on the horses’ experiences, to provide an overall grade of Quality of Life and are tailored to chronically ill or geriatric horses.

**Abstract:**

Equine Quality of Life (QoL) is an important concern in decision making in veterinary medicine and is especially relevant for chronically ill or geriatric horses towards the end of their lives. To our knowledge, there is no currently available QoL assessment tool for chronically ill or geriatric horses that assesses equine QoL defined as the horse’s evaluation of their life. However, tools exist to assess equine welfare in different contexts. Hence, the aims of this study were to analyse how equine welfare, QoL, well-being and happiness assessment tools label, define and operationalise the concepts and to discuss the tools’ suitability to assess equine QoL in the context of end-of-life decisions for chronically ill or geriatric horses. Fourteen articles were found through a systematic literature search, describing ten equine welfare assessment tools and one approach to integrating equine QoL in veterinary practice that suggests QoL assessment parameters. We discuss that some welfare assessment tools have the potential to support the development of a QoL assessment tool informing decisions towards the end of horses’ lives if they are adjusted to focus on the horses’ experiences, to provide an integration into an overall QoL grade and are tailored to chronically ill or geriatric horses.

## 1. Introduction

Equine Quality of Life (QoL) is an important concern in decision making in veterinary medicine [1,2,3,4]. Despite its relevance, however, there is no one universally accepted definition of equine QoL [5,6]. We base the definition of equine QoL on definitions of QoL for animals in general [7,8,9,10,11]. Drawing from these, equine QoL is defined as an individual’s subjective evaluation of their life, which in turn is assumed to involve a balance of positive and negative affective states (and cognitive evaluations where possible) [7,8,9] over an extended period of time [10,11]. Relying on the theory of core affect [12], affect can be summarised as “a subjective experience that varies in pleasantness or unpleasantness (valence) as well as activation (arousal)” [13] (p. 62). QoL is rated on a bipolar continuum meaning that a horse can have a poor, neutral or good QoL and all states in between [14,15]. Different factors can influence an animal’s affective state and therefore its QoL, such as fulfilment of needs, health, social relationships, control and choice [7,9]. How much an individual’s QoL is influenced by something also depends on the individual’s preferences, personality and experiences in their life so far [7,8,9,16]. Taylor and Mills discuss some of the challenges associated with attempting to assess animal QoL as an individual’s subjective evaluation of their life because this evaluation cannot be accessed directly and the extent to which animals cognitively evaluate their life is unclear [8]. However, it is not required for the horse to be thinking about their life to have a QoL according to our working definition; it is sufficient for the horse to have “a sense of well-being” [8] (p. 61), which may or may not include cognitive evaluations of their life experiences [8]. McMillan summarises this as the “affective and cognitive (to the degree that the animal can form such a cognitive construct) assessment that an animal makes of its life overall, of how its life is faring” [14] (p. 193).

There are two reasons for considering the horse’s affective balance *over time* for QoL assessments: the horse’s perspective on their life and the requirement for a decision about euthanasia by the responsible human(s). Regarding the horse’s perspective on their life, there is great uncertainty over which time periods are actually relevant to the horse’s evaluation of their life as McMillan and Yeates also argue for animals in general [15]. We would neither expect a horse to live purely in the moment where past experiences and affective states play no role in their current sense of how their life is faring, nor would we expect a horse to be cognitively evaluating their whole past and potential future life to arrive at an overall QoL evaluation for their life. Instead, the answer most likely lies somewhere in between.

In light of the uncertainty about the relevant time period from the horse’s perspective, deciding about treatment or euthanasia of chronically ill or geriatric horses requires humans to take responsibility for this decision. Such a decision always comes with an implicit idea of what it means for a horse to have a good life. This will likely also consider the (possible) future of the horse [17,18]. Defining QoL as the balance of affective states over an extended period of time intends to separate QoL from short-term affective states that would not be considered enough to base decisions about treatments or euthanasia on them. Imagine for example a horse that is scared by a plastic bag. The horse will experience a momentary state of stress and fear, but unless this happens frequently, it would not be considered an impairment of the horse’s QoL and certainly not be relevant for a decision about euthanasia. Which time period is of relevance for a decision cannot be answered without the context of the specific decision.

### 1.1. QoL in Comparison to Welfare

QoL shares similarities with animal welfare and some see them even as identical in meaning [19,20]. Others emphasise that the only difference between them is the relevant time frame: Whereas animal welfare refers to an individual’s state at a point in time, QoL refers to longer time periods [10,21,22]. In addition to welfare, there are other related concepts, e.g., (subjective) well-being and happiness, where a distinction from QoL is not always made [15]. McMillan and Yeates [15] compared the use of this terminology and found that different terms for what they subsume under the umbrella term of animal *Well-being* are often used synonymously and interchangeably in the literature. Nevertheless, they also found a clear tendency for QoL (together with happiness and subjective well-being) to refer to a type of *psychological well-being* that requires conscious processing by the individual [15]. In contrast, welfare (and well-being) is categorised as including both *psychological and physical well-being*, which means that a physical influence can affect welfare without any conscious processing by the respective individual [15]. This would, for example, mean that the welfare of a horse can be affected by a health condition such as a small tumour, because the horse’s health is compromised, while the horse’s QoL can be unaffected as long as the tumour has no influence on the horse’s affective states.

Looking at the historical development, welfare assessment predominantly focused on groups of animals and the avoidance of suffering, whereas QoL highlights the relevance of the individual and of positive states [8]. However, with more recent developments of ‘positive animal welfare’ [23,24] and an emphasis on the relevance of individual differences in welfare assessment [16], the lines between QoL and welfare are increasingly blurred. We therefore work with an understanding of QoL as a continuation of animal welfare with a strong emphasis on the individual and their subjective experience over longer time periods.

### 1.2. Two Functions of QoL Assessment near the End of Horse’s Lives

Next to other functions of QoL assessment in veterinary care, such as measuring the impact of treatment on QoL, QoL is especially relevant for chronically ill or geriatric horses in palliative care and end-of-life decisions when the horse is not expected to recover from their illness and the reconstitution of health is not the primary goal of treatment anymore. According to Shearer “[p]alliative care addresses the treatment of pain and other clinical signs to achieve the best quality of life regardless of disease outcome“ [25] (p. 330). This results in a shift in the primary goal of treatment from curing the horse to caring for the horse [26]. In this context, QoL is a criterion for when it is the right moment and, in the horse’s best interest, to end a horse’s life, because the horse’s QoL cannot be sustained at or improved to an acceptable level and any further interventions are deemed unlikely to change that. Assessing the QoL of a chronically ill or geriatric horse then has two practical functions: (1) to enable sustaining or improving the horse’s QoL as much as possible and (2) to inform a decision about euthanasia of the horse.

In order to be able to improve a horse’s QoL, ideally, one would first assess the horse’s QoL, try to understand why the QoL is compromised [27], introduce changes accordingly and re-evaluate whether the intended improvement was achieved. However, it is not always essential to arrive at an overall evaluation of QoL to bring about improvements. It would be sufficient to assess a subset of the total parameters that could inform changes to improve this particular aspect of the horse’s QoL without integrating the parameters into an overall grade for the horse’s QoL. For example, if a horse is experiencing pain, analgesics provide the possibility to improve the horse’s QoL without weighing the level of pain against other factors of QoL, such as the horse’s possibility to interact with conspecifics. However, if the goal of the QoL assessment is to inform a decision about whether or not to euthanise a horse due to poor QoL, it is not sufficient to assess a range of parameters without any weighing or prioritising of these parameters.

For a QoL assessment in the context of a life-or-death decision, some form of overall judgement of the horse’s QoL is necessary, even if one parameter is so severely compromised that other aspects of the horse’s life cannot compensate for it; for example, if a horse suffers from severe pain that does not respond to treatment. In that case, the overall judgement is straightforward and implicit in judging the severe pain as critical for the horse’s QoL. In less clear-cut cases, such as the slow deterioration of chronically ill horses’ states over time, the different aspects of a horse’s life need to be somehow integrated into a final judgement to allow the conclusion that the horse’s life is not worth living anymore due to poor QoL. The human’s judgement of a horse’s QoL would ideally be identical to the horse’s own appraisal of their life. Since human proxies have been found to not always accurately assess another human’s QoL [28,29,30,31,32,33,34], it is to be expected that humans’ evaluations of a horse’s QoL will deviate from this ideal. In addition to that, not only the current QoL of a horse is relevant for a decision, but also whether any interventions could still improve a horse’s QoL to a level where euthanasia is not required. Thus, predictions about future QoL states [17,18] are an important aspect of a decision about euthanasia. Furthermore, an assessment of the owner’s willingness and resources to execute potential interventions to improve the horse’s QoL is also part of the decision-making process.

These aspects will also factor into the decision about which level of QoL is deemed acceptable for a particular horse, a highly contextual decision for which humans remain responsible even if an assessment tool provides an overall grade of the horse’s QoL. A QoL assessment tool can inform, but not in itself determine, this decision, e.g., by providing a means to assess change over time in QoL and by ensuring that no relevant aspects are overlooked.

### 1.3. Types of Parameters in QoL and Welfare Assessment Tools

QoL assessment tools typically consist of questions directed at the veterinarian and the owner or caregiver of an animal as human proxies [35,36,37,38,39,40]. Such tools can therefore encourage veterinarians and owners to reflect on and discuss their observations and assessments related to an animal’s QoL, and thereby reduce the risk of overlooking or underestimating important aspects relevant to the animal. Assessment parameters, also for welfare assessment tools, are typically categorised into horse-based parameters, resource-based parameters, and management-based parameters [41]. Horse-based parameters are assessed at or with the horse (such as through behavioural observations or health assessment), whereas resource-based parameters are concerned with resources (such as the size of the box or floor type) available to the horse and management-based parameters are concerned with decisions made by human caregivers that influence the horse’s life, such as time on pasture, type and time of exercise [41]. Which types of parameters are most suitable for QoL assessment (1) to enable sustaining or improving the horse’s QoL as much as possible and (2) to inform a decision about euthanasia of the horse will be addressed in the following.

#### 1.3.1. Parameters for QoL Improvements

To be able to use QoL assessment to improve a horse’s QoL, it is important to discriminate between indicators that reflect QoL (“indicators”) and factors of QoL (“factors”) that influence and therefore predict QoL (such as availability of food or space) [8,18]. To assess a horse’s current QoL, indicators reflecting a horse’s QoL are therefore preferable over factors of QoL, because the former reflect the actual state of the horse, whereas the latter are only assumed to influence a particular horse in a certain way. However, as Browning analogously points out for assessment of subjective welfare [42], assessing factors of QoL plays an important role in improving a horse’s QoL, since they inform where changes are possible that could result in a better QoL.

Whether or not a parameter in an assessment tool is an indicator or a factor of QoL or welfare depends on the definition of what is being assessed. Health or more precisely, the physical state of the horse, provides an example of this. There are different definitions of health [43,44,45], which is why we used the term physical state to separate it from mental states and reflections thereof (e.g., behaviour) when analysing the assessment tools. When welfare is defined as consisting of physical and mental health, physical health is an indicator directly reflecting the horse’s welfare. For a QoL assessment following the definition of QoL as the animal’s subjective evaluation of their life, physical health can be both, a factor or an indicator [8,18,46]. The mental state of a horse is likely to be negatively influenced by severe lameness (physical health as a factor), whereas mental states such as prolonged stress due to, for example, missing contact with other horses have the potential to affect the horse’s immune system and manifest as physical health issues (physical health as an indicator). Therefore, to evaluate a horse’s QoL, it is not sufficient to assess the horse’s physical health; it is important to also consider how much it is affecting the horse mentally (to the extent that this is possible) and whether health problems or physiological parameters are actually a reflection of the horse’s mental state.

Since our working definition of equine QoL assumes that a horse’s subjective evaluation of its life is based on a balance of positive and negative affective states, parameters assessing a horse’s mental state are most likely to function as accurate indicators of that horse’s QoL. Hausberger et al. [27] point out the importance of behaviour for the assessment of the subjective experience of the horse, which they equate with welfare, and state that “behavior is a core aspect of welfare, being the interface between the organism and its environment” [27] (p. 15). Yeates [22] also discusses the importance of behaviour in assessing an animal’s QoL. Thus, parameters that might be considered indicators reflecting QoL could be found in the analysed assessment tools in the form of behavioural parameters intended to indirectly assess aspects of the mental state of the horse.

#### 1.3.2. Indicators of Overall QoL

To arrive at an overall evaluation of QoL for the second function of a QoL assessment, informing euthanasia decisions, equine QoL defined as the subjective evaluation of an individual’s life by that individual would ideally be assessed by an indicator that directly reflects the horse’s evaluation of their life or the balance of positive or negative affective states over time. For welfare as a subjective state, Browning calls such an indicator a “whole-animal measure” [42] (p. 176). In that sense, the integration of all possible factors influencing a horse’s QoL “has already taken place within the mind of the animal” [42] (pp. 176–177) and the outcome, the horse’s QoL, can be assessed via this single measure. To be consistent with our definitions of indicators and factors of QoL, we will refer to such a parameter as a *whole-animal indicator*. Browning [42] discusses cognitive bias testing, neuroimaging and Qualitative Behavioural Assessment (QBA) as possible candidates for such whole-animal indicators. Cognitive bias tasks have been developed for animals in general and for horses to assess long-term emotional states, also called moods [47,48,49,50,51]. As a type of neuroimaging, measurements of the resting-state quantitative EEG power spectrum of a horse have been demonstrated to have potential for assessing the horse’s “chronic welfare state” [52] (p. 2), whereby welfare refers to the “subjective experience” [52] (p. 2) of the horse. QBA aims to assess an animal’s emotional state through detailed observation of the animal by an experienced observer who scores the animal based on predefined or freely chosen descriptors such as anxious, playful, relaxed, etc. [53]. QBA has been studied in the context of horses, e.g., for assessing short-term effects [54,55] and the human–horse relationship [56]. However, the validity of the method, especially for adult horses, has not yet been unequivocally established [27].

In the absence of a validated whole-animal indicator of a horse’s QoL, an assessment of QoL according to our working definition would need to reflect the balance of negative and positive affective states over time. To capture that balance, observations over a longer time or repeated evaluations of short-term states would be a way forward.

When the assessment of QoL is based on multiple parameters (factors or indicators), these parameters need to be integrated into an overall evaluation of the horse’s QoL as a basis for a decision about euthanasia. Such an overall evaluation should be centred around the horse’s affective or mental states as the representation of the horse’s QoL.

### 1.4. Aim and Research Question

To our knowledge, there is no currently available assessment tool that explicitly evaluates the QoL of chronically ill or geriatric horses, whereas there are different welfare assessment tools for horses [57,58,59,60,61,62,63,64,65,66,67]. Due to the terminological and conceptual ambiguity, it is not clear, however, whether existing horse welfare, well-being or happiness assessment tools provide a useful approach for evaluating QoL. Hence, the aim of this paper is to discuss existing equine welfare, QoL, well-being and happiness assessment tools to inform decisions about the end of chronically ill or geriatric horses’ lives. To achieve that, we address the following research question: How do equine welfare, QoL, well-being and happiness assessment tools label, define and operationalise the concepts they aim to assess?

On the basis of a literature search, we discuss the results with regards to the tools’ suitability to assess equine QoL in the context of end-of-life decisions for chronically ill or geriatric horses focussing on aspects derived from the two practical functions: (1) to enable sustaining or improving the horse’s QoL as much as possible and (2) to inform a decision about euthanasia of the horse. The tools’ suitability to assess QoL according to our working definition depends on the following aspects: the definitions of the concepts assessed, the types of parameters included, whether and how an overall grade for the concept assessed is included, and whether the assessment aims at a snapshot or is an assessment over time. Our working definition of equine QoL requires a strong focus on the affective or mental state of the horse and how the horse evaluates their life. How tools define the concepts they assess influences their choices and relative importance of the parameters. The types of parameters of the assessment tools are relevant for sustaining or improving a horse’s QoL and should ideally include indicators reflecting the horse’s QoL, such as behavioural parameters. Whether the tools provide an overall grade of QoL, welfare, happiness or well-being is relevant to inform a decision about euthanasia of the horse. How this overall grade is arrived at shows the relative importance of parameters and whether the horse’s affective or mental state is given priority as required by our working definition of QoL. The duration or frequency of assessment is of interest for our research context since our working definition of QoL requires the assessment of affective states over time as opposed to a momentary recording.

## 2. Materials and Methods

**Figure 1:**
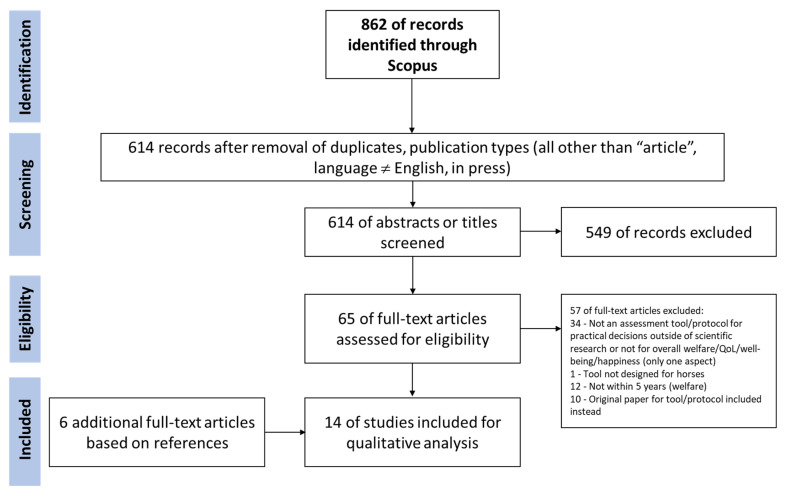
Flow chart illustrating the selection of studies included in the review.

A systematic literature search was conducted to identify publications on assessment tools for equine QoL, welfare, well-being or happiness for the purpose of analysing how these publications label, define and operationalise the concepts. The literature search was conducted following the Preferred Reporting Items for Systematic Reviews and Meta-Analyses (PRISMA) guidelines [68] with minor adjustments concerning the inclusion of additional articles (see Figure 1).

### 2.1. Data Sources and Searches

The literature search was conducted using the electronic database Scopus (Elsevier, Amsterdam, The Netherlands; https://www.scopus.com, accessed on 16 December 2021). Scientific peer-reviewed articles on equine QoL, welfare, well-being or happiness assessment tools published after 2005 were identified using a combination of terms for horses (horse*, equine, equus), well-being (Quality of Life, welfare, well-being, wellbeing, happiness) and assessments (measurement*, assessment*, evaluation*, tool*) in title, abstract or keywords. The search was conducted in November 2021 with the following query string: (TITLE-ABS-KEY (horse*) OR TITLE-ABS-KEY (equus AND ferus AND caballus) OR TITLE-ABS-KEY (equus AND caballus) OR TITLE-ABS-KEY (equine*)) AND (TITLE-ABS-KEY (quality AND of AND life) OR TITLE-ABS-KEY (welfare) OR TITLE-ABS-KEY (well-being) OR TITLE-ABS-KEY (wellbeing) OR TITLE-ABS-KEY (happiness)) AND (TITLE-ABS-KEY (assessment*) OR TITLE-ABS-KEY (measurement*) OR TITLE-ABS-KEY (evaluation*) OR TITLE-ABS-KEY (tool*)) AND PUBYEAR > 2005.

Identified records were organised using Microsoft^®^ Excel^®^ 2019 (Microsoft Corporation, Redmond, WA, USA) and Zotero v5.0.96.3. (Corporation for Digital Scholarship, Vienna, VA, USA, https://www.zotero.org/, accessed 12 March 2022).

The selection of publications for full-text analysis as well as the actual full-text analysis and data extraction was conducted by the first author (M.L.) following the procedure outlined in Figure 1. A random sample of abstracts was analysed independently by M.L. and C.D. to discuss and clarify the in- and exclusion criteria.

Search parameters for the electronic database were set to exclude (a) conference papers, reviews, book chapters, books, conference reviews, notes, short surveys/mini reviews, editorials, letters, errata, reports, data papers, retracted papers, abstract reports, business articles, undefined publications and (b) publications not written in English.

Based on abstracts and titles, publications were excluded if they (c) were not (also) focused on horses older than 1 year or (d) did not describe the assessment of overall QoL, welfare, well-being or happiness and were only focused on one aspect (such as pain or stress) unless the authors of the assessment tool declare that aspect to be equivalent to welfare, well-being, happiness or QoL.

By means of the full-text analysis, publications were included if they were (i) peer-reviewed articles, that were (ii) written in English and if (iii) they described or used a QoL, welfare, well-being or happiness assessment tool or protocol for horses above 1 year of age, designed to inform practical decisions outside of scientific studies (such as adjusting husbandry conditions, making decisions about veterinary treatments for a horse etc.). The intention behind this was to include only tools with a level of complexity and preparation required (e.g., to train the horses) that would be manageable outside of research contexts. Assessment tools and protocols are defined here as a set of pre-defined, structured questions, criteria or parameters. If publications described or used the same QoL, welfare, well-being or happiness assessment tool or protocol, only the (iv) original publication that contained the complete tool or protocol was included. Assessment protocols focused on welfare were further time-restricted and included if they fell within a time limit of 5 years (November 2016–November 2021) to limit the higher number of publications on welfare assessments to the latest tools that are more recently applied in practice and more likely to reflect the latest understandings of welfare. Well-being, QoL or happiness assessment tools were not further time limited because of the limited number of relevant publications found.

For peer-reviewed articles using a tool first described outside the framework of peer-reviewed journals, the original publication of the tool was included as well, if it was available in English. In addition to that, other assessment tools were included if they were referenced in full-text analysed papers, fell within the limit of 5 years (welfare tools/protocols) and fulfilled the same inclusion criteria outlined above (i–iv).

### 2.2. Data Extraction

The following information was extracted and summarised from the publications included in the study:

- The key term for the concept the tool addresses (QoL, welfare, well-being, happiness or a different one).
- Definitions or descriptions of the concept the tool addresses.
- Whether and how many behavioural parameters, horse-based parameters of physical states of the horse, resource-based and management-based parameters are included.
- How parameters are integrated into an overall grade.
- Intended length and frequency of assessment.
- Intended context for the assessment tool.

## 3. Results

A total of 862 records were identified through Scopus (see also Figure 1). After removal based on duplication, publication type and language (non-English), 614 records remained. After screening abstracts and titles, 549 records were excluded. The remaining 65 articles were read in full. There were 8 articles that remained in the final analysis, 57 articles were excluded based on the criteria outlined above whereby 34 were not an assessment tool for practical decisions outside of scientific research or not for overall welfare/QoL/well-being/happiness, 12 were outside the 5-year time frame, for 10 the original papers of the tool were included instead and 1 tool was not designed for horses.

An additional 6 articles were identified through references, so that 14 articles were analysed, describing 11 different QoL or welfare assessment protocols. The additional articles consisted of three non-peer-reviewed documents detailing welfare assessment tools [66,67,69], two peer-reviewed articles presenting welfare assessment tools [59,61] and one peer-reviewed article presenting a tool to aggregate welfare parameters into scores [62]. The additional articles had not been found through the original search but only through references in full-text analysed papers, since they were either not peer-reviewed [66,67,69], used the term equid (instead of equine or horse) in abstract, title and keywords [59,62] or were listed as a review and therefore not found in the initial literature search but were eventually included because of the welfare assessment approach they suggest [61].

### 3.1. Overview of Assessment Tools and Analysed Publications

Eleven assessment tools were identified in the literature search. As displayed in Table 1, the tools were designed for use in different contexts [59,62], such as welfare assessment for working horses [58,60], free-roaming horses [61] or horses on farms [57,64,66,67,70], as well as legislative or code-of-practice compliance [63,65] and veterinary practice [17]. Some of the tools were explicitly developed for the context of equine or equid welfare organisations [58,59,60,62], for example, to decide about the allocation of resources for welfare improvements [62].

### 3.2. Labels and Definitions Used in Assessment Tools

With the exception of one protocol [17], the tools identified in the literature search focus on the assessment of equine (or equid) welfare (see Table 2). The terms ‘quality of life’ and ‘well-being’ are mentioned by some welfare assessment tools, but not as the focus of the tool [57,58,59,70]. Parker and Yeates [17], who identify six steps involved in QoL-based decision making for equine patients (see Table 3), describe QoL as being focused on the mental experience of the horse. They use the term welfare in some cases interchangeably with QoL but also highlight differences, namely, an association of welfare with negative states and the focus on the individual for QoL [17]. They also once mention the term “well-being” [17] (p. 244); however, they do not further discuss or distinguish the concept.

Not all welfare assessment tools provide an explicit definition of welfare (see Table 2). A commonly recurring theme is welfare as a “multidimensional concept” [66] including aspects such as physical and mental health [59] without necessarily providing a hierarchy of the different aspects of welfare. Parameters of welfare risk (mainly resource- and management-based) and parameters of welfare state (mainly animal-based) are distinguished in some of the welfare assessment tools [53,61,62,64,66]. The protocol presented by Harvey et al. [61] is based on the Five Domains Model, e.g., [10,82], and outlines the importance of the mental state of the individual animal by equating it to the overall welfare state. All other parameters are then assessed in the light of how they affect the individual’s mental state [61]. DuBois et al. [63] and Raw et al. [59] also reference the Five Domains Model, whereas others quote the Welfare Quality^®^ approach as a basis or an influence for their welfare assessment approach [57,64,66,67,69,70]. The Welfare Quality^®^ approach was developed within the 6th EU framework programme and considers four principles, ‘Good feeding’, ‘Good housing’, ‘Good health’ and ‘Appropriate Behaviour’, as relevant for animal welfare and translates these into twelve related criteria for on-farm welfare assessment [66].

### 3.3. Types of Parameters

The assessment tools were analysed with regards to whether they include behavioural parameters of the horse, whether they include parameters of the physical state of the horse and whether they assess parameters related to resources and management. In addition, the number of parameters in each category was counted. Most (9/11) of the assessment tools measure the behaviour of equines, but 2 out of the 11 assessment tools do not include direct behavioural parameters of welfare [63,65] (see Table 4). The number of resource/management-based parameters differs (range: 1–68) between the welfare assessment tools; the Standardised Equine-Based Welfare Assessment Tool (SEBWAT), for example, includes only one such parameter, but 32 horse-based parameters [58], whereas the welfare assessment tool by DuBois et al. [63] includes 68 resource- and management-based parameters and only 6 horse-based parameters. Whether a tool includes more horse-based (range: 2–38) or more resource/management-based parameters can be understood as a first indication of the main focus of the tool. In none of the assessment tools does the number of behavioural parameters exceed the number of parameters of the physical state of the horse.

In the only analysed article focusing on QoL assessment, Parker und Yeates [17] provide a six-step protocol for how to make decisions in a veterinary context while including the assessment of the horse’s QoL. They give examples of behavioural parameters as well as parameters to assess the horse’s health or physical state and resource- or management-based parameters but do not include a list of parameters suitable for analysing the numbers of parameters in each category [17].

### 3.4. Integration into One Overall Welfare or QoL Grade

Six of the eleven assessment protocols provide no integration into an overall welfare grade for individuals or groups of horses (SEBWAT [58], EARS [59], *Unnamed-I* [60], Horse welfare assessment protocol (HWAP) [64], ‘Swedish official protocol’ [65] and *Unnamed-III* [63]). Three welfare assessment protocols provide instructions on how to integrate results into an overall score or grade on the level of the farm or a group of horses, but not for individual horses (Animal Welfare Indicators welfare assessment protocol for horses (AWIN) [66], ‘Welfare monitoring system’ [69] and Welfare Aggregation and Guidance Tool (WAG) [62]). When less than 10 horses are being assessed, the AWIN protocol recommends assessing the compliance of each individual horse with all 12 criteria, which in turn consist of multiple indicators [66]. Similarly, the WAG includes, as a first step, scores and grades for each individual horse for each of five welfare categories (health, behaviour, working conditions, living environment and nutrition), before these scores are further combined to obtain group-level grades [62].

The welfare assessment tool by Harvey et al. [61] is based on the Five Domains Model, e.g., [10,82], and was the only tool found to recommend a procedure for integrating different parameters of welfare for an individual horse into two grades, one for *welfare compromise* and one for *welfare enhancement.* The most negative physical impact on the mental state of the horse represents the overall welfare compromise, whereas the overall welfare enhancement is determined by opportunities for self-motivated rewarding behaviours and their utilisation, and a “cautious judgement of the degree of ‘*positive affective engagement*’” [61] (p. 16). The integration should be conducted based on scientifically informed knowledge about the impact of physical Domains 1–4 onto the mental state of the horse (Domain 5) [61]. Numerical scores are avoided, however, to prevent the aggregation of scores [61]. In addition, Harvey et al. recommend a separate grading of “welfare alerting indices” [61] (p. 6) to inform about whether interventions for the assessed individual are required because the individual is at risk of future welfare compromises.

Parker and Yeates [17] also advise against using numerical scores for the overall QoL of horse patients when there are disagreements between owners and veterinarians about how to proceed, and instead suggest questions directed at the owner of the horse to encourage them to reflect upon the overall QoL of their horse.

### 3.5. Duration and Frequency of Assessment

The welfare assessment tools record welfare at a point in time. Some tools recommend monitoring changes over time, for example, to assess the impact of interventions by horse welfare organisations to improve equine welfare in a certain area or country [58]. Viksten et al. [64] repeated the welfare assessment using the HWAP after 16 or 25 days to assess the reliability of the assessment process during the development of the protocol, but this repetition is not a regular part of the protocol. A repetition of the assessment for an individual horse is not required to complete any of the welfare assessments. In addition to that, the need for an assessment to be completed in a short amount of time for practical reasons is often highlighted [58,59,63].

For the SEBWAT, it is made explicit that its “results are recorded at one brief moment in time” [58] (p. 16–17) and are, according to the authors, therefore not representing the horse’s Quality of Life. The only QoL assessment protocol among the analysed papers describes that “QOL assessment should be an ongoing process” [17] (p. 247) that requires adjusting veterinary treatment.

## 4. Discussion

### 4.1. The Two Practical Functions of QoL Assessment

This paper addressed the research question of how equine welfare, QoL, well-being and happiness assessment tools label, define and operationalise the concepts. In the following, we discuss the results with regard to the assessment tools’ suitability to assess equine QoL in the context of palliative care and end-of-life decisions for chronically ill or geriatric horses. We focus on aspects derived from the two practical functions of a QoL assessment in this context: (1) to enable sustaining or improving the horse’s QoL as much as possible and (2) to inform a decision about euthanasia of the horse. As outlined in the introduction, we base the definition of equine QoL on definitions of QoL for animals in general [7,8,9,10,11] and define equine QoL as an individual’s subjective evaluation of their life, which in turn is assumed to involve a balance of positive and negative affective states (and cognitive evaluations where possible) [7,8,9] over an extended period of time [10,11]. As we discuss in the following sections, the analysed assessment tools require some adjustments before they are suitable to fulfil both of the outlined functions of QoL assessments for chronically ill or geriatric horses.

### 4.2. Identified Tools and Their Definitions of Welfare and QoL with Regards to Their Suitability to Assess QoL

The following paragraphs discuss how the identified assessment tools label and define their concepts and whether the concepts align with our working definition of QoL. Of the eleven tools that were analysed for this paper, ten focus on the welfare of horses [57,58,59,60,61,62,63,64,65,66,67,69,70], and one protocol suggests a six-step process for integrating equine QoL assessment into the context of veterinary decisions for equine patients [17]. This discrepancy in numbers was expected and emphasises the motivation for this study to analyse whether the numerous available horse welfare assessment tools might mitigate a need for designated QoL assessment tools for horses. No well-being or happiness assessment tool was identified in this study; hence, well-being or happiness assessment tools and the concepts of well-being and happiness will not be part of the following discussion, which focuses on welfare and QoL.

Parker and Yeates [17] suggest parameters for equine QoL and how they might be assessed, as well as six steps for integrating QoL into veterinary decision making. They state QoL is about “mental experiences” [17] (p. 244) and reference McMillan’s account of animal QoL, which regards affective states as central for QoL [9], and which we also refer to for our definition of equine QoL. The protocol is not in itself a fully developed QoL assessment tool but can nevertheless inform our discussion since it also addresses the inclusion of equine QoL in the context of veterinary care for horses.

When it comes to definitions of welfare in publications about the welfare assessment tools, Harvey et al.’s [61] definition of welfare as the horse’s subjective mental experience, based on the Five Domains Model, is similar to our working definition of equine QoL. DuBois et al. [63] also reference the Five Domains Model and state that “welfare assessments must account for the subjective experiences of the animal” [63] (p. 38). The majority of the welfare assessment tools, however, do not have this strict focus on the mental experience of the horse and provide somewhat similar definitions or descriptions of welfare. A common theme is to describe welfare as “multidimensional” [64] (p. 59) [66] (p. 9) and via different aspects and combinations of those aspects that are relevant for welfare such as mental and physical health [59,60,62,66], coping and harmony with the environment [66], behavioural needs [65,70], freedom from suffering [59] as well as natural lives [62,65]. The WAG, for example, describes that natural life, physical well-being and mental state are included in animal welfare [62]. This is in line with the common account of animal welfare as comprising elements of all those three aspects [42,46]. However, this account of welfare does not provide information on how to weigh different aspects of welfare, especially when they are in conflict with each other, since “[i]f each of these factors is seen as equally primary in welfare, then there is no reason that one should win out over another” [42] (pp. 38–39).

This is in contrast to our working definition of QoL as the horse’s evaluation of their life, which is assumed to consist of the balance of the horse’s affective states over an extended period of time. Here all other aspects, such as the horse’s health or the way they live their life e.g., with regards to naturalness, are only relevant in so far as they affect the horse’s subjective experience and subsequent evaluation of their life (or are a reflection thereof). We refer the reader to Section 4.4.2. for a discussion of how the individual nature of QoL can be addressed in indirect assessments through human proxies. As we discuss in the following, the different definitions of welfare and QoL are also reflected in how the tools assess these concepts, which affects whether they are suitable for QoL assessments to (1) improve a horse’s QoL as much as possible and (2) inform a decision about euthanasia of a horse.

### 4.3. The Assessment Tools’ Suitability for QoL Assessment to Improve a Horse’s QoL

In the following, we will discuss whether the analysed assessment tools are suitable for QoL assessment to sustain or improve a horse’s QoL based on how they operationalise their concepts with regards to indicators and factors of QoL.

Parker and Yeates [17] speak of measurements of input when discussing their suggestion for equine QoL assessment in the context of veterinary care. Inputs refer to everything that influences the horse’s life, such as resources and management provided by humans, interactions with other horses, but also aspects of the horse itself such as their health [17]. In that sense, inputs are factors that are *assumed* to influence a horse’s QoL. Therefore, they can inform about possible problems and risks for QoL [17], but it is important to keep in mind that the assumptions about the relevance of these factors for a particular horse might be inaccurate. Some of the welfare assessment tools also emphasise that resource- and management-based as well as some animal-based parameters measure welfare risks but not the actual welfare state of a horse [41,61,62,64,66] whereby the welfare risks can inform about potential future problems.

Ideally, QoL assessment tools should include parameters intended to measure the horse’s affective or mental states such as behavioural parameters. The analysed welfare assessment tools use less behavioural than health- (physical state), resource- and management-based criteria (see Table 4), suggesting a higher priority of physical state and living conditions. However, the lower number of behavioural parameters can also be due to the limited availability of validated indicators of affective or mental states (for recent reviews find, e.g., [85,86,87]). Assessing the validity and reliability of the parameters in the analysed assessment tools was not part of the present work (find, e.g., Hausberger et al. [27] for a detailed discussion of the SEBWAT and the AWIN tool) but is a crucial aspect to consider before using the tools or when developing a new one. In addition to that, the importance of criteria is not only defined by how many of them there are in an assessment tool but rather by how they are weighed against each other when an overall welfare grade is determined.

In summary, the measurement of both, influences on the horse’s QoL (factors) and reflections of their QoL (indicators), are relevant to improving a horse’s QoL. Hence, parameters evaluating behaviour, physical state, resources and management should be included in a QoL assessment tool, as is the case in most but not all of the analysed welfare assessment tools. In addition to that, the criteria’s (assumed) relationship to equine QoL (factor or indicator) needs to be considered to give them the appropriate weight, especially for arriving at an overall QoL grade.

### 4.4. The Assessment Tools’ Suitability for the Assessment of Overall QoL to Determine When to Euthanise a Horse

In the following, we discuss how the tools operationalise their concepts and address whether the analysed assessment tools are suitable for the assessment of overall QoL to determine when to euthanise a horse. In order to arrive at an overall evaluation of the horse’s QoL, it would be ideal to assess a whole-animal indicator as part of the QoL or welfare assessment, which represents how the horse experiences and evaluates their life. None of the analysed welfare assessment tools suggest assessing the welfare or QoL via one of two possible candidates for a whole-animal indicator, neuroimaging [52] or cognitive bias tasks [47,48,49,50,51]. This is not surprising, given the complexity of the assessment and analysis and the time required to train horses to take part in the tests, especially in the case of cognitive bias tasks. For the welfare assessment tools analysed in this study, time was often a crucial factor and horses were previously unknown to the assessor and assessed without prior training of the horse. The complexity of the two parameters would also limit their applicability to assess the QoL of horses in a context where assessors, which could be the horse’s owner, had more time or knew the horse well.

Some of the welfare assessment tools did include a different parameter that, however, can be understood as a whole-animal indicator: a parameter for the horse’s “general attitude” [60] (p. 3) [62] (p. 6) [59] (p. 5) [58] (p. 7) or a short version of QBA [66]. The general attitude parameter is similar to a QBA assessment because it also relies on an observation of the horse by the (trained) assessors who summarise their overall impression in this parameter. If cautiously assessed, the general attitude of the horse in a QoL assessment can supplement a decision about euthanising a horse. Given the questions regarding the validity of QBA [27] and the potential for bias depending on who is assessing the horse, QBA or the determination of the general attitude of the horse, should not be the only elements in a decision about euthanasia. In addition to that, the general attitude of the horse might be short-term and could change quickly depending on what causes it. Furthermore, there are many other factors relevant in a decision about euthanising a horse, for example, whether the horse’s QoL can still be improved.

#### 4.4.1. Relevance of Time in QoL Assessment

In the absence of a whole animal indicator, capturing the balance of affective states over time would be required according to our working definition of QoL. Observations over a longer time or repeated assessments of short-term states could achieve this. Currently, the analysed welfare assessment tools do not require repeated assessments; however, this could be relatively easily implemented for a QoL evaluation.

Changes in QoL over time will also require repeated assessments. Parker and Yeates explain this in their six-step protocol for QoL-based decisions for equine patients, by pointing out that QoL assessment is “an ongoing process” [17] (p. 247) during a horse’s veterinary care that should lead to constant adjustments in the treatment of the horse to account for side effects and adverse consequences of the treatment. In the case of chronically ill or geriatric horses, the assessment of QoL remains a continuous part of palliative care, which only ends with the patient’s death [25,26].

This need for considering extended time periods or repetitions of QoL assessments, comes at the expense of practicability. It is frequently mentioned for the welfare assessment tools that the time needed to complete them should be short for practical reasons [58,59,63]. This is also due to the nature of the studies in which the tools were used, where participants could not spare a lot of time (e.g., in the cases of working equids). Parker and Yeates [17] also emphasise that any QoL assessment tool needs to be adequate for practice and advocate a flexible approach where different assessment approaches might be used in different contexts. A possible way to address this could be a two-level approach as in the AWIN tool [57,66]. In the AWIN tool, the second, more extensive assessment is only conducted under certain conditions and when problems are detected during the more concise and quicker to perform first-level assessment [57,66]. A similar approach could be useful in the screening of QoL either for multiple horses or for regular checks for a particular horse, where a more extensive assessment including resource- and management-based parameters takes place if animal-based parameters suggest a compromised QoL.

Another possibility to enable repeated or more time-intensive QoL assessments in practice would be to provide horse owners with validated assessment tools they can apply. However, the ability of owners to fulfil this task and the required expertise should be carefully considered.

In summary, in order to assess QoL according to our working definition, the equine welfare assessment tools would require adjustments or repetitions of the assessment to capture long-term mental states or a balance of mental states over time as opposed to short-term states of the horse.

#### 4.4.2. Integration of Multiple Parameters into Overall QoL

Multiple parameters (factors or indicators) need to be integrated into an overall evaluation of an individual horse’s QoL as a basis for a decision about euthanasia. None of the welfare assessment tools provide a way to aggregate the scores into one overall grade for an individual horse; although, some tools provide integration strategies for groups of horses (AWIN [66], ‘Welfare monitoring system’ [69] and WAG [62]). This makes sense, because it was not the aim of these tools to assess individual horses but rather, for most of them, to provide information about welfare levels for groups or populations of horses. However, the lack of an integration of criteria into one overall grade for an individual horse also means that their suitability for QoL assessments for a chronically ill or geriatric horse in a clinical context and for related end-of-life decisions is limited.

Out of the welfare assessment tools analysed for this study, the Five Domains Model comes closest to an integration of parameters in line with our working definition of QoL [61]. It provides a way of aggregating criteria into two grades for an individual horse, a welfare compromise grade and a welfare enhancement grade, whereby the maximum possible welfare enhancement is also determined by the welfare compromise [61]. This means that the amount to which positive experiences can compensate for negative ones is limited. This model actively avoids numerical scores to avoid the impression that grades and scores can be calculated, and underlines the necessity for reflection on the welfare parameters and what they mean for a particular horse [61]. Parker and Yeates [17] similarly suggest avoiding numerical scores for (some) horse owners and rather recommend questions that encourage the horse owner to reflect on the overall QoL of their horse, such as “‘Would you like to live your horse’s life?’ and ‘Does your horse have more good days than bad days?’” [17] (p. 247).

Browning [42] criticises that the integration in the Five Domains Model is “done informally and based primarily on the knowledge and intuition of the assessor/s” [42] (p. 182). This, however, is not only a potential problem but can also be considered a strength. It allows for the adjustment of the weighing of criteria to the individual horse by those who know the horse well, which, given the highly subjective nature of QoL, might be more appropriate than weighing the factors and indicators based on a species-norm [5]. Some horses, for example, might place a high value on continuous contact with other horses while for others, a quiet resting place at night might be more valuable. On the other hand, this non-objective approach invites bias in the weighing of the individual aspects, which can lead to an over- or underestimation of the QoL of a horse. There is a danger for trivialising problematic states for a horse if a QoL assessment is overly individualised. For example, a behavioural expression of discomfort through aggression could be described as part of a horse’s personality when it might actually result from chronic pain [88] or social problems due to mismanagement of group housing [89,90]. Owners of geriatric horses have also been found to underestimate health issues in their horses [91,92]. Incorporating more than one person’s opinion into the assessment may help to reduce biases or lack of expertise. Hence, weighing parameters of QoL based on the individual horse is in line with our working definition of QoL, but should be performed carefully and be well-informed. Out of the analysed welfare assessment tools, the welfare assessment tool based on the Five Domains Model [61] provides a promising candidate for this.

### 4.5. The Different Tools and Their Suitability for QoL Assessment Based on the Aspects Discussed

Based on our working definition of QoL as an individual’s subjective evaluation of their life, Table 5 provides an overview of suitable and unsuitable aspects of the analysed tools with regards to assessing QoL. The following aspects are taken into consideration: the definition of the concepts used, the parameters included in the tools, the repetition of assessments to capture long-term states, and the integration of the criteria into an overall grade. This is not an evaluation of the quality or the usefulness of the assessment tools as such. As outlined in Table 1, the contexts and goals the tools were designed for differ from the goals and context we are analysing them for. Hence, whatever their suitability for the assessment of equine QoL according to our working definition is, we do not mean to imply that the tools are not well-designed or not suitable for their intended purpose.

In summary, out of those assessment tools analysed for this paper, the one built upon the Five Domains Model seems most promising for an assessment of QoL according to our working definition because all parameters are considered as relevant in relation to their influence on the individual’s mental state [61]. This resembles the core idea to reflect the individual’s perspective as key to QoL. The welfare assessment tool analysed here is focused on non-captive wild horses so that it is not directly applicable to chronically ill or geriatric horses in human care [61]. Nevertheless, a combination of the tool’s integration approach and the justification for parameters in the tool with parameters from other welfare assessment tools for horses in human care could be a way forward for a QoL assessment tool for chronically ill or geriatric horses. Parker and Yeates [17] provide ideas for equine QoL assessment in veterinary practice and the Equid Assessment, Research and Scoping (EARS) tool is set up as a repository of questions that can be combined into different welfare assessment tools; among others, a tool for equids in sanctuaries has been developed that could inform a QoL assessment tool for chronically ill or geriatric horses [59].

### 4.6. Limitations of the Study

The restriction to a limited number of welfare assessment tools from recent years cannot provide a full overview of possible approaches to welfare assessment of horses. Including only tools that aim to provide an overall welfare assessment and excluding those papers that only present one indicator could have resulted in the exclusion of potential whole-animal indicators. For some papers, it was not straightforward to judge, whether they constituted a presentation of one of the multiple welfare indicators or whether the authors considered their welfare indicator the only one necessary. The publication by Kim and Cho [93] about infrared thermography to measure eye temperature in the context of equine welfare assessment presents an example of such a case. However, as outlined above, for a QoL assessment to fulfil both its functions, an assessment of overall QoL, but also of QoL factors, is required, so that a single whole-animal indicator could not achieve this.

The original tool was not always accessible in English in full detail (question catalogue of the EARS [59], ‘Swedish official protocol’ [65]), so that information might be missing, e.g., concerning overall grades. This also reduces the comparability of the numbers in Table 4, since it cannot be guaranteed that parameters are split up in the same manner, potentially resulting in different numbers between different tools, when effectively the same aspects of a horse’s life are measured.

The quality of the tools with regards to the validity and reliability of the criteria they use was not assessed in this study. In addition, we did not discuss in detail who is using the tools and the required level of expertise. Most of the analysed welfare assessment tools explicitly require the assessor to be trained [57,58,59,60,63,66,67]. Parker and Yeates [17] discuss the benefits and drawbacks of including the horse owner in the assessment of QoL. The intended user is a necessary consideration for the development of an equine QoL assessment tool.

## 5. Conclusions

In order to be suitable for QoL assessments for chronically ill or geriatric horses, the currently available welfare assessment tools that were analysed for this paper would require some adjustments. When it comes to informing end-of-life decisions regarding a chronically ill or geriatric horse, most welfare assessment tools do not sufficiently satisfy the requirements of prioritising the subjective mental experience of the horse, of integrating criteria into one overall grade or of focusing on long-term as opposed to momentary states. Related limitations have been found for some QoL assessment tools for dogs and cats [35,94]. The welfare assessment tool for free-roaming horses based on the Five Domains Model, which defines welfare as the subjective experience of an individual and evaluates and integrates outcomes of the multiple parameters based on this [61], is a promising candidate for an equine QoL assessment tool. However, this tool would require an adjustment to chronically ill and geriatric horses and their long-term mental states, as well as the establishment of one overall grade. The different welfare assessment tools provide a range of parameters that are potential factors of QoL, which is a strength regarding the second function of a QoL assessment in the context of palliative care, monitoring QoL and its changes in response to interventions aimed to sustain or improve a horse’s QoL.

For the future, further parameters focused on the mental state of the horse and parameters specifically for chronically ill or geriatric horses [5] should be developed and included in QoL assessment tools. Since an equine QoL assessment in the context of palliative care aims to fulfil two functions, informing improvements of QoL over time or in response to interventions and informing a decision about euthanasia of a horse, it is important to include assessments of resources and management as well as parameters for overall QoL as much as possible.

The analyses of the included welfare assessment tools and the protocol for QoL assessment also show that there is no strict division between welfare and QoL assessment, analogous to the overlap between the concepts of QoL and welfare. This highlights the importance of providing a definition of the targeted concept of any assessment tool.

## Figures and Tables

**Table 1:** Analysed welfare and QoL assessment tools with the context they were used in or were designed for, the publications the analyses were based on (primary source) and additional publications using the tool that was identified in the literature search, but not included in the analysis.

Tool	Primary Source	Context	Also Used in
**EARS|**Equid Assessment, Research and Scoping tool	Raw et al., 2020 [59]	Catalogue of questions to build welfare assessment protocols for different contexts such as working equids or feral equids	[71,72,73]
***Unnamed tool-I***	Fröhlich et al., 2020 [60]	Welfare assessment for working horses in Fiji to inform strategies for welfare improvement	-
***Unnamed tool-II***	Harvey et al., 2020 [61]	10-stage welfare assessment protocol for free-roaming horses	-
**WAG|**Welfare Aggregation and Guidance Tool	Kubasiewicz et al., 2020 [62]	Aggregation of welfare parameters (of the EARS tool) into scores to identify differences in welfare trends between groups to inform allocation of resources	[72]
**SEBWAT|**Standardised Equine-Based Welfare Assessment Tool	Sommerville et al., 2018 [58]	Equine welfare assessment for working equids in low- and middle-income countries	-
***Unnamed tool-III***	DuBois et al., 2018 [63]	On-farm welfare assessment for Canadian equine industry designed to evaluate farms’ adherence to the National Farm Animal Care Council’s Equine Code of Practice [74]	[75]
**HWAP|**Horse welfare assessment protocol	Viksten et al., 2017 [64]	On-farm horse welfare assessment for detection of welfare issues for informed improvements	[41,76]
**‘Swedish official protocol’**	Hitchens et al., 2017 [65]	Protocol to assess compliance with Swedish and European Union (EU) animal welfare legislation	[41]
**AWIN|**Animal Welfare Indicators welfare assessment protocol for horses	Dalla Costa et al., 2016 [57]	On-farm welfare assessment of single-stabled horses (<5 years old)	[77,78,79,80,81]
	AWIN, 2015 [66]	Welfare assessment for single-stabled horses over 5 years old at farm level to compare similar management and production systems	
***Unnamed tool-IV***	Parker and Yeates, 2012 [17]	Suggestions for an approach to Quality-of-Life assessments for equine patients	-
**‘Welfare monitoring system’|**Welfare monitoring system: assessment protocol for horses	Sanmartín Sánchez et al., 2020 [70]	Welfare assessment at Spanish Army Breeding Centre with a modified version of ‘Welfare monitoring system’	-
	Wageningen UR Livestock Research, 2011 [67]	On-farm welfare assessment for horses	
	Wageningen UR Livestock Research, 2012 [69]	Calculation of scores for ‘Welfare monitoring system’	

**Table 2:** Definitions and descriptions of equine or animal welfare in analysed publications of equine welfare assessment tools including their basis on other approaches such as the Welfare Quality^®^ principles of Good feeding, Good housing, Good health and Appropriate Behaviour, e.g., [57,66] and the Five Domains Model with its prioritisation of the animal’s mental state as their welfare state, e.g., [10,61,82].

Tool	Primary Source	Definitions and Descriptions of Equine or Animal Welfare
**EARS|**Equid Assessment, Research and Scoping tool	Raw et al., 2020 [59]	“An animal’s ability to experience complete mental and physical health, and be able to live without suffering in an environment provided or adapted by human beings” (p. 2) What is considered ‘good welfare’ changes with the context Reference to Five Domains Model and the importance of factors that influence internal or mental state of the animal
***Unnamed tool-I***	Fröhlich et al., 2020 [60]	No explicit definition Pain, fear and health issues given as issues that can “lead to compromised welfare” (p. 2) Behaviour as a useful “indicator of how welfare issues are making an animal feel” (p. 14)
***Unnamed tool-II***	Harvey et al., 2020 [61]	Historically: welfare focused on “‘fitness’ (physical states)” (p. 3) Contemporary understanding of welfare: focus on “‘feelings’ (mental experiences or affective states)” (p. 3) Welfare as the subjective mental experience of an individual’s life Welfare “as a property of individuals, belonging to species considered [to] have the capacity for both pleasant (positive) and unpleasant (negative) mental experiences, a capacity known as sentience” (p. 3) Based on the Five Domains Model: four domains reflecting “physical/functional domains of welfare; ‘nutrition’, ‘environment’, ‘health’ and ‘behaviour’, and a fifth domain of mental state (affective/mental experience)” (p. 4) Domain 5 represents the welfare state and is influenced by affective consequences of animal-based measurements of Domains 1–4
**WAG|**Welfare Aggregation and Guidance Tool	Kubasiewicz et al., 2020 [62]	“Ultimately, good welfare can be viewed as the state in which an animal experiences a ‘good life’ (…)” (p. 2) Includes: physical wellness, mental state, natural life Meaning can depend on the observer and context
**SEBWAT|**Standardised Equine-Based Welfare Assessment Tool	Sommerville et al., 2018 [58]	Status of the animal, best represented by animal-based health and behaviour parameters Distinguished from Quality of Life, since “results are recorded at one brief moment in time” (p. 16–17)
***Unnamed tool-III***	DuBois et al., 2018 [63]	“welfare assessments must account for the subjective experiences of the animal” (p. 38) Intended to assess both the state of the animal and their living conditions Reference to the Five Domains Model, which focuses on mental states
**HWAP|**Horse welfare assessment protocol	Viksten et al., 2017 [64]	Welfare as multidimensional: physical and mental health, including comfort, absence of hunger, thirst, disease and fear, and the animal’s own experience of their environment Reference to Welfare Quality^®^ approach, which considers Good feeding, Good housing, Good health and Appropriate Behaviour, as relevant for animal welfare
**‘Swedish official protocol’**	Hitchens et al., 2017 [65]	No definition provided (not the original paper for the tool) Mentioning of “peaceful and natural intake of feed and water” (p. 1243), “need for social contact” (p. 1242) and “good animal health” (p. 1243)
**AWIN|**Animal Welfare Indicators welfare assessment protocol for horses	Dalla Costa et al., 2016 [57]	No definition of horse welfare provided Based on Welfare Quality^®^ research, which considers Good feeding, Good housing, Good health and Appropriate Behaviour, as relevant for animal welfare
	AWIN, 2015 [66]	Multidimensional concept A state of complete mental and physical health and of harmony with environment State of animal as regards its attempts to cope with its environment, referencing Broom [83] Based on Welfare Quality^®^ principles: Good Housing, Good Feeding, Good Health, Appropriate Behaviour Animal-based indicators to find out about the actual state of the animal; resource- and management-based parameters to identify (welfare) risk factors
**‘Welfare monitoring system’|**Welfare monitoring system: assessment protocol for horses	Sanmartín Sánchez et al., 2020 [70]	No explicit definition Different aspects relevant to welfare are mentioned such as negative experiences also through husbandry conditions, behavioural needs and “positive welfare effects” (p. 138) Reference to Welfare Quality^®^, which considers Good feeding, Good housing, Good health and Appropriate Behaviour, as relevant for animal welfare
	Wageningen UR Livestock Research, 2011 [67]	No explicit definition of welfare Based on Welfare Quality^®^, which considers Good feeding, Good housing, Good health and Appropriate Behaviour, as relevant for animal welfare Assessment is multidisciplinary Welfare of “an animal at that time” (p. 3) should be assessed via multiple parameters Emphasis on health and behaviour
	Wageningen UR Livestock Research, 2012 [69]	Based on Welfare Quality^®^ approach, which considers Good feeding, Good housing, Good health and Appropriate Behaviour, as relevant for animal welfare

**Table 3:** Definitions and descriptions of equine or animal QoL in analysed publications of equine QoL assessment tools.

Tool	Primary Source	Definitions and Descriptions of Equine or Animal QoL
***Unnamed tool-IV***	Parker and Yeates, 2012 [17]	QoL as “philosophical matter” (p. 244), meaning it encompasses ethical issues as well as requiring scientific knowledge about horses [84] QoL is about mental experiences, referencing McMillan who defines QoL as consisting of affective states [9] Health can be relevant for QoL due to resulting unpleasant feelings but QoL also includes “experiences, such as enjoyment, frustration and anxiety” (p. 244) Description of Five Freedoms (freedom from hunger and thirst; discomfort; pain, injury and disease; to express normal behaviour; and from fear and distress) and Five Opportunities for welfare (opportunity for selection of dietary inputs; for control of the environment; for pleasure, development and vitality; to express normal behaviour; for interest and confidence) Interchangeable use of welfare and QoL but explanation that QoL avoids the negative connotations of welfare and is more individualistic

**Table 4:** Welfare and QoL assessment tools and number of horse-based (HB), resource-based (RB) and management-based (MB) parameters for horses (> 1-year-old), integration approach, assessment frequency and time required for the assessment.

Publications	HB (^#^ of Parameters)		RB + MB (^#^ of Parameters)	Integration into Overall Grade for Individual Horse	Time Per Horse and/or Frequency of Assessment
	Behavioural Parameters	Physical State			
**EARS|Equid Assessment, Research and Scoping tool** based on Raw et al., 2020 [59]					
[59]	X (6)	X (32)	X (39)	no	Time per horse or frequency not indicated
***Unnamed tool-I*** based on Fröhlich et al., 2020 [60]					
[60]	X (1)	X (9)	X (24)	no	Time per horse not indicated Assessment conducted once
***Unnamed tool-II*** based on Harvey et al., 2020 [61]					
[61]	X (8)	X (20)	X (29)	yes	Not explicitly stated (parameters given are examples, not a final protocol yet)
**WAG|Welfare Aggregation and Guidance Tool** based on Kubasiewicz et al., 2020 [62]					
[62]	X (2)	X (6)	X (12)	partially	Time per horse or frequency not indicated Tool intended for monitoring changes over time
**SEBWAT|Standardised Equine-Based Welfare Assessment Tool** based on Sommerville et al., 2018 [58]					
[58]	X (4)	X (28)	X (1)	no	5–10 min/animal If required, repetition to assess impact of interventions for equid populations (not necessarily for individual equids)
***Unnamed tool-III*** based on DuBois et al., 2018 [63]					
[63]	O	X (6)	X (68)	no	Every horse assessed once Overall time per farm on average 144 min+/− 15 min, also depending on number of horses
**HWAP|Horse welfare assessment protocol** based on Viksten et al., 2017 [64]					
[64]	X (1)	X (17)	X (31)	no	10–12 min/horse + RB- and MB parameters Assessment was repeated after 16 or 25 days to assess reliability
**‘Swedish official protocol’** based on Hitchens et al., 2017 [65]					
[65]	O	X (2)	X (43)	no	Time per horse not indicated Repetition of assessment under certain circumstances (e.g., if checked previously because of a complaint)
**AWIN|Animal Welfare Indicators welfare assessment protocol for horses** based on Dalla Costa et al., 2016 [57] and AWIN, 2015 [66]					
[57]	(see line below)	(see line below)	(see line below)	partially	First level: 5 min/horse Second level: 11–20 min/horse No repetition required
[66]	X (6; 5 ^#^)	X (14; 15 ^#^)	X (5; 4 ^#^)	partially	5 min per horse for first-level assessment 11–25 min per horse in second-level assessment No repetition required
***Unnamed tool-IV*** based on Parker and Yeates, 2012 [17]					
[17]	X	X	X	yes	QoL assessment as an ongoing process (in the context of decisions about veterinary treatment)
**‘Welfare monitoring system’|Welfare monitoring system: assessment protocol for horses** based on Sanmartín Sánchez et al., 2020 [70], Wageningen UR Livestock Research, 2011 [67], Wageningen UR Livestock Research, 2012 [69]					
[70]	X (4)	X (15)	X (28; +3 ^#^)	no	Time per horse not indicated All horses assessed once
[67]	X (1)	X (22)	X (10)	no	15–20 min per horse
[69]	n/a	n/a	n/a	no	n/a

X = parameters included; n/a = not applicable; O = parameters not included; ^#^ for horses in groups.

**Table 5:** Suitable and unsuitable aspects of the different assessment tools for assessing QoL according to our working definition based on the tool’s definition of welfare or QoL, parameters used, the integration of parameters into an overall grade and assessment of long-term vs. short-term states.

Suitable Aspects for QoL Assessment	Unsuitable Aspects for QoL Assessment
**EARS|Equid Assessment, Research and Scoping tool** based on Raw et al., 2020 [59]	
Importance of mental states for welfare mentioned Catalogue of questions that allows for tools for specific contexts Includes behavioural parameters and parameter ‘general attitude’ could be understood as a whole-animal indicator	Equal importance of physical and mental health according to their definition of welfare No integration of criteria into one overall grade for an individual horse Unclear whether repetition is required according to final tool
***Unnamed tool-I*** based on Fröhlich et al., 2020 [60]	
No explicit definition of welfare, but acknowledgement of importance of behaviour Behavioural parameter (‘general attitude’) could be understood as a whole-animal indicator	Only one behavioural criterion No repetition of assessment intended Focused on working horses; however, these often have health problems and might therefore be similar in some regards to chronically ill horses No integration into one overall grade for an individual horse
***Unnamed tool-II*** based on Harvey et al., 2020 [61]	
Welfare as mental state of the horse, importance of subjective experience of the individual Integration of criteria into 2 grades for an individual horse Criteria and their integration into overall grades based on effects on mental state of the horse	Wild horses as target population, not chronically ill or geriatric horses in human care Not a finished tool but suggestions for parameters to include No integration into one overall grade for an individual horse
**WAG|Welfare Aggregation and Guidance Tool** based on Kubasiewicz et al., 2020 [62]	
Provides an approach to integrate parameters into five grades for an individual Includes behavioural parameters and parameter ‘general attitude’ could be understood as a whole-animal indicator	Intends to monitor (changes in) welfare trends between groups, not focused on individual horses No clear prioritisation of mental state of horse based on definition or integration of criteria No integration into one overall grade for an individual horse
**SEBWAT|Standardised Equine-Based Welfare Assessment Tool** based on Sommerville et al., 2018 [58]	
Focus on the state of the horse and therefore animal-based criteria Behavioural parameter (‘general attitude’) could be understood as a whole-animal indicator	Only one behavioural parameter in the tool Lack of resource- and management-based criteria to inform improvements for QoL Fast assessment suggests it records only momentary state No repetition for individual horses intended No integration into one overall grade for an individual horse
***Unnamed tool-III*** based on DuBois et al., 2018 [63]	
Highlights importance of subjective experience of the individual for welfare assessment	No behavioural parameters, main focus on resources and management due to focus on adherence to code of practice No integration into one overall grade for an individual horse No repetition required by the tool
**HWAP|Horse welfare assessment protocol** based on Viksten et al., 2017 [64]	
Welfare as multidimensional and including, among other aspects, subjective experience	No clear prioritisation of subjective experience or mental state of animal according to definition of welfare Only one behavioural parameter (occurrence of unwanted behaviour), which is unlikely to be a whole-animal indicator No integration into one overall grade for an individual horse No repetition required by the tool
**‘Swedish official protocol’** based on Hitchens et al., 2017 [65]	
Resource and management parameters can inform potential improvements	Missing definition of welfare Resource and management focused Repetition only required in some cases No integration into one overall grade provided for an individual horse
**AWIN|Animal Welfare Indicators welfare assessment protocol for horses** based on Dalla Costa et al., 2016 [57] and AWIN, 2015 [66]	
Mental health as important for welfare, but among other aspects Different behavioural parameters, including QBA, which could be understood as a whole-animal indicator 2-level approach could be useful for screening of multiple horses	No prioritisation of mental state of individual over other aspects of welfare such as physical health according to definition of welfare No integration into one overall grade for an individual horse No repetition required
***Unnamed tool-IV*** based on Parker and Yeates, 2012 [17]	
Focused on QoL Similar definition of QoL to our working definition, importance of mental state	Not a finished tool for QoL assessment yet
**‘Welfare monitoring system’|Welfare monitoring system: assessment protocol for horses** based on Sanmartín Sánchez et al., 2020 [70], Wageningen UR Livestock Research, 2011 [67], Wageningen UR Livestock Research, 2012 [69]	
Emphasises importance of behaviour and health Includes behavioural parameters	No prioritisation of mental state of individual over other aspects of welfare according to definition of welfare Only one behavioural parameter in original tool [67] No integration into one overall grade for an individual horse No repetition required

## Data Availability

Full list of publications excluded during the selection process of the systematic literature search is available from the corresponding author on request.
